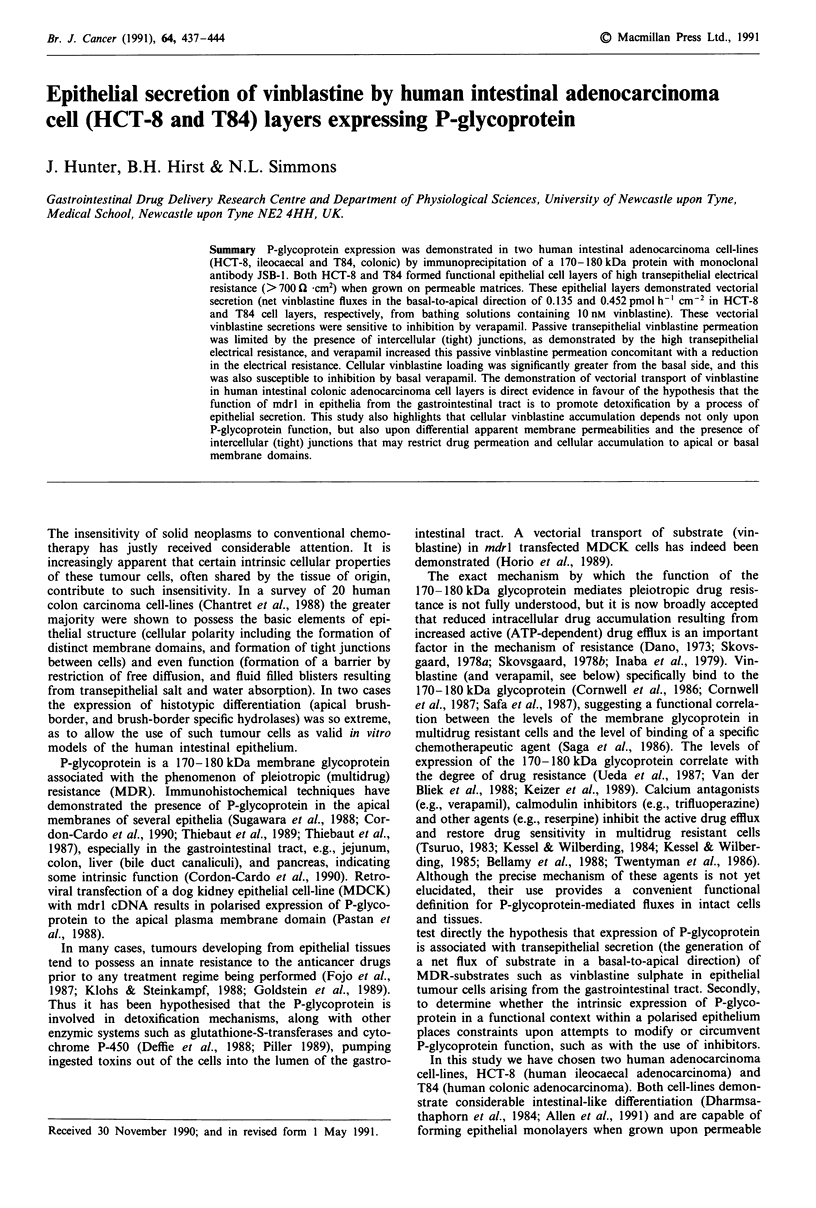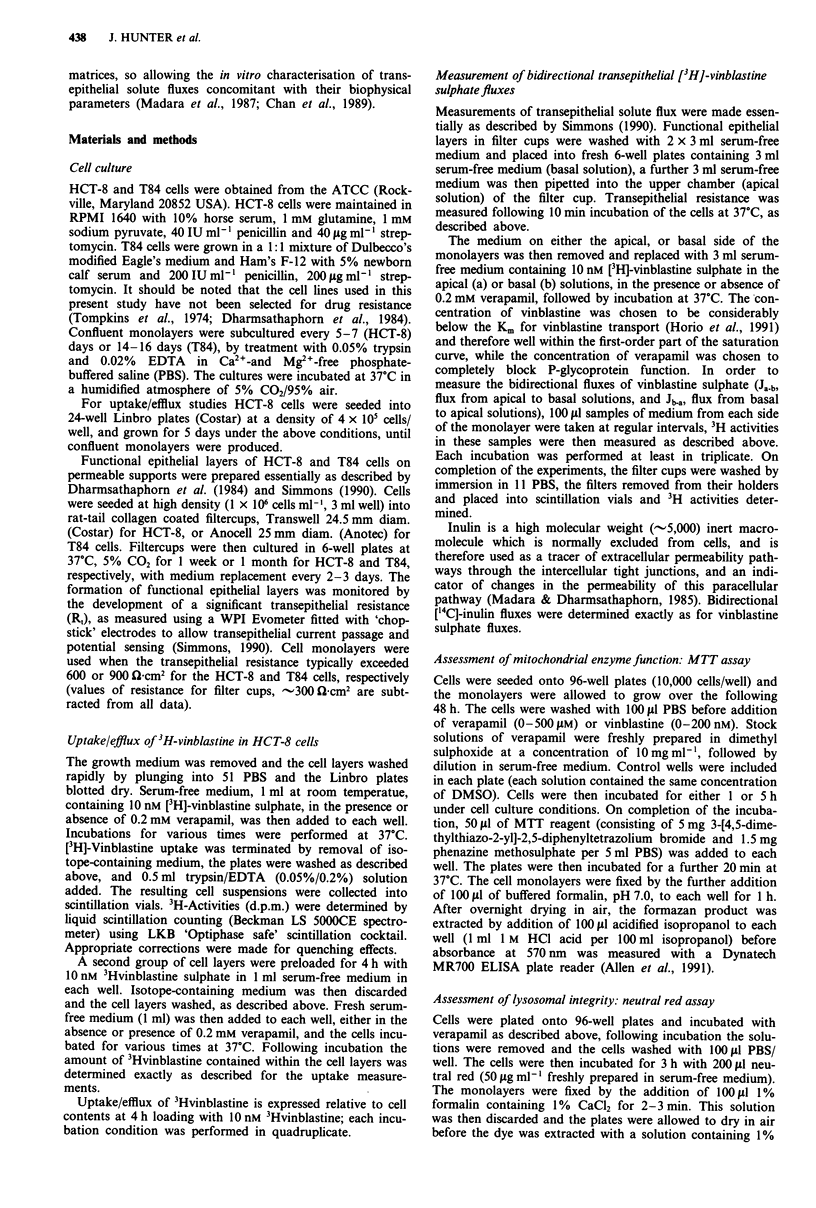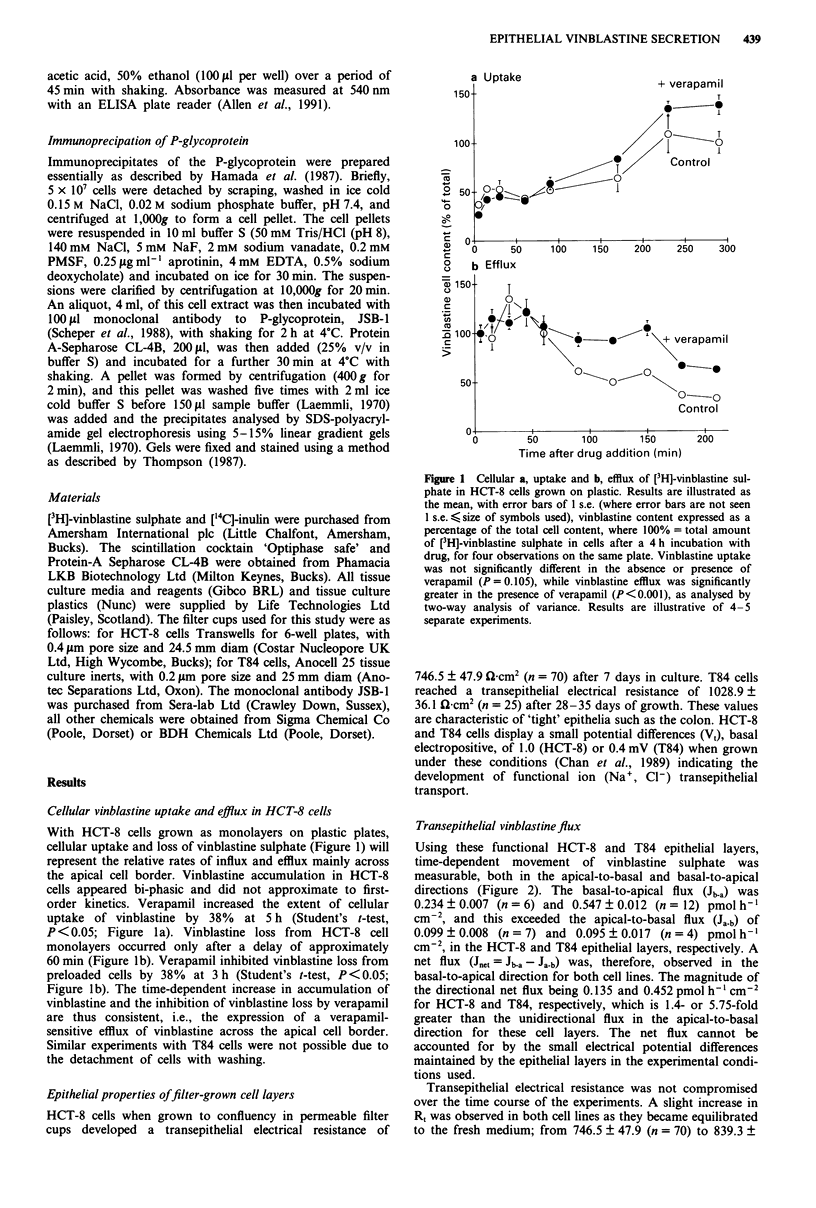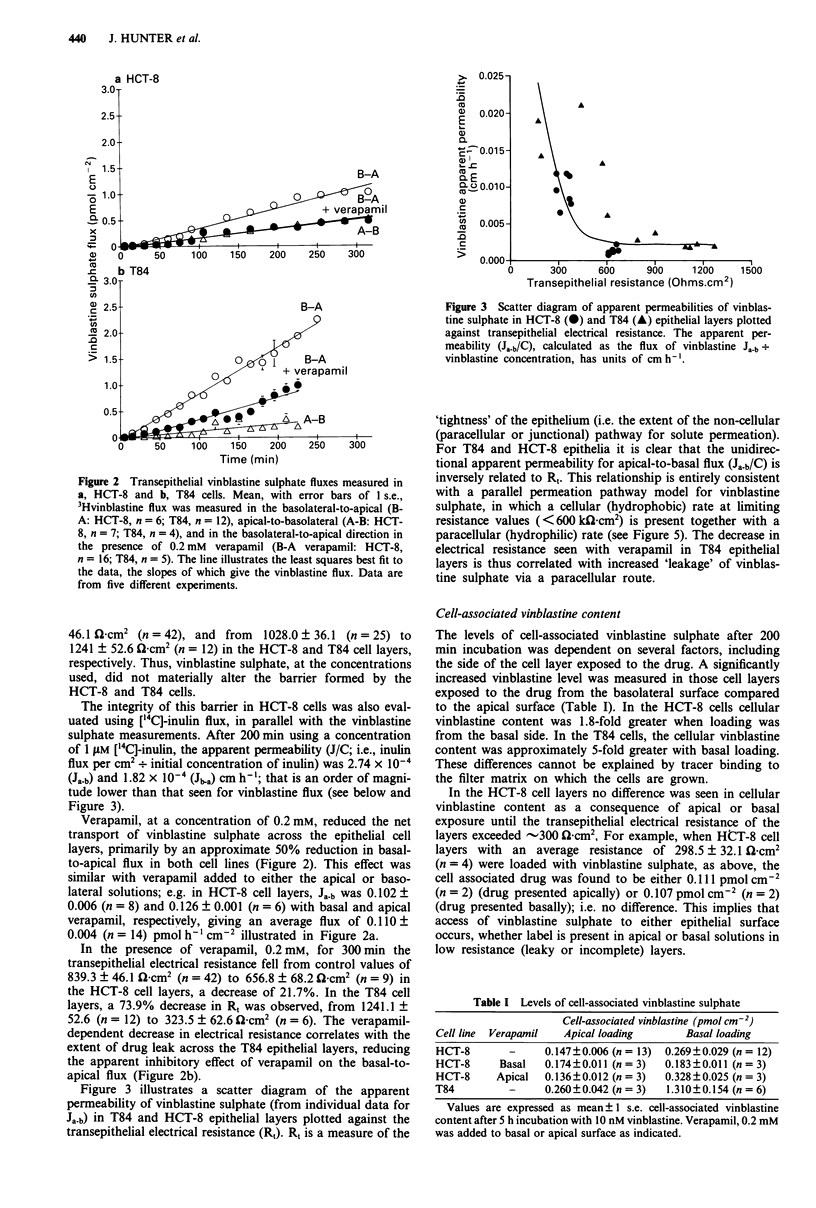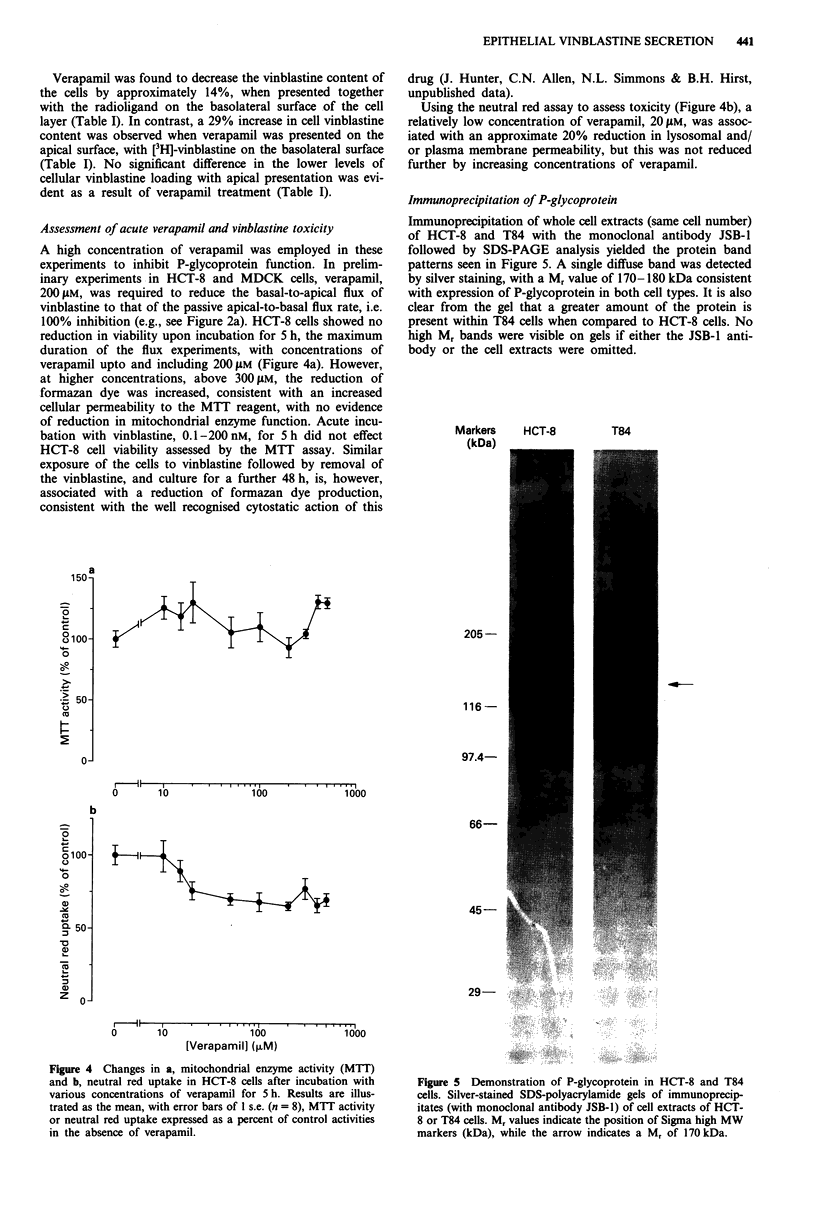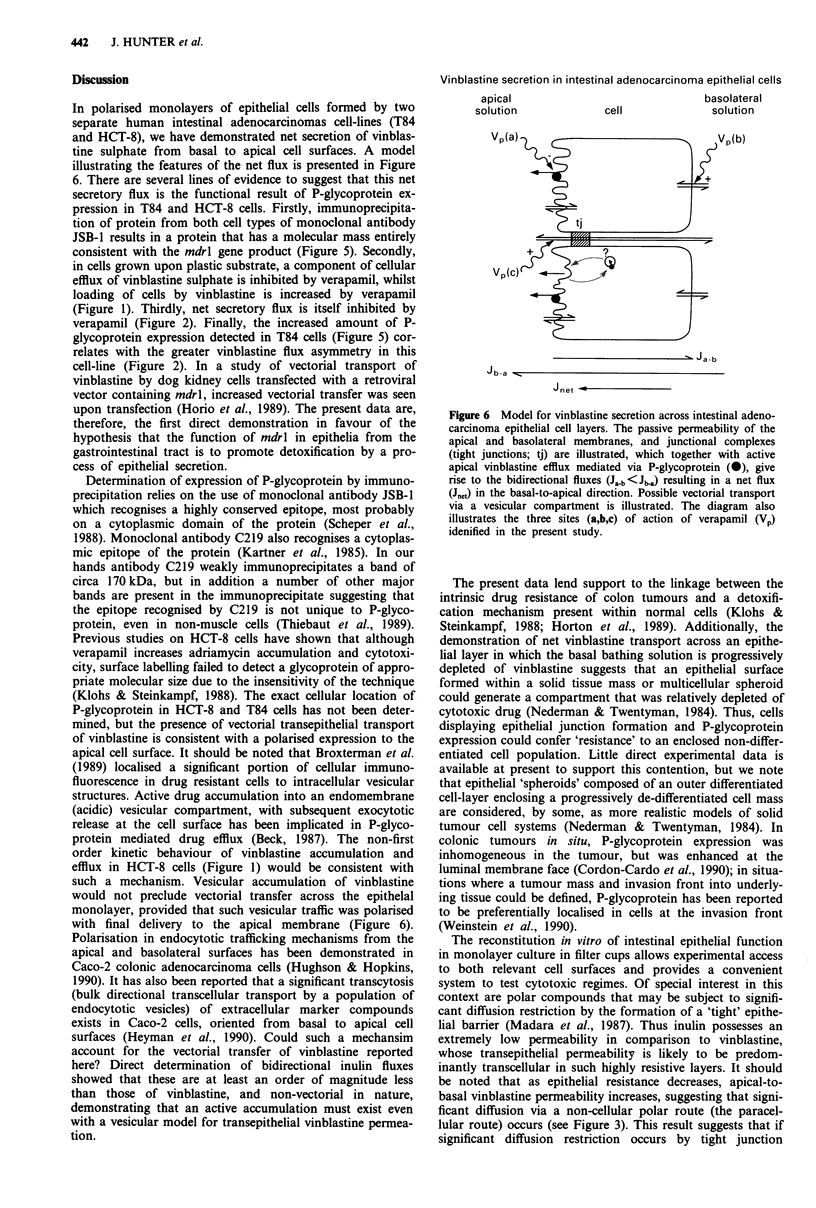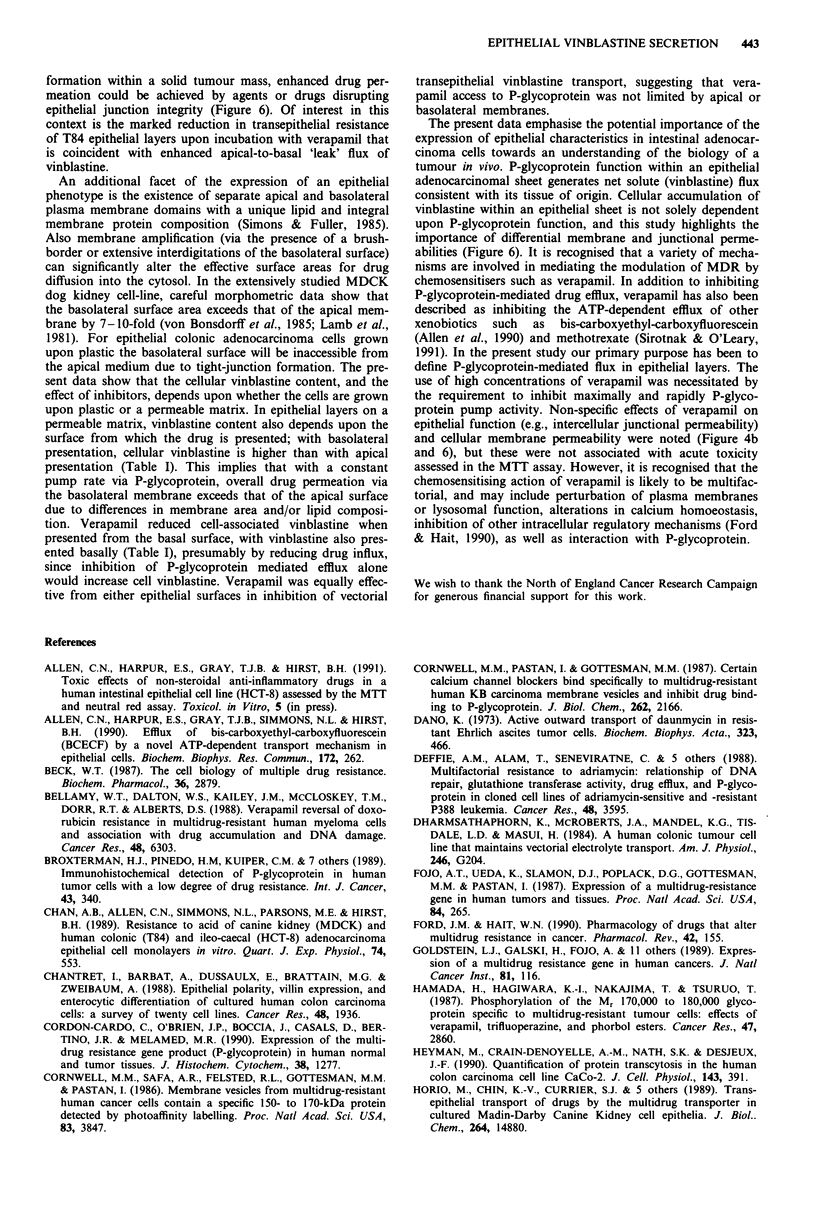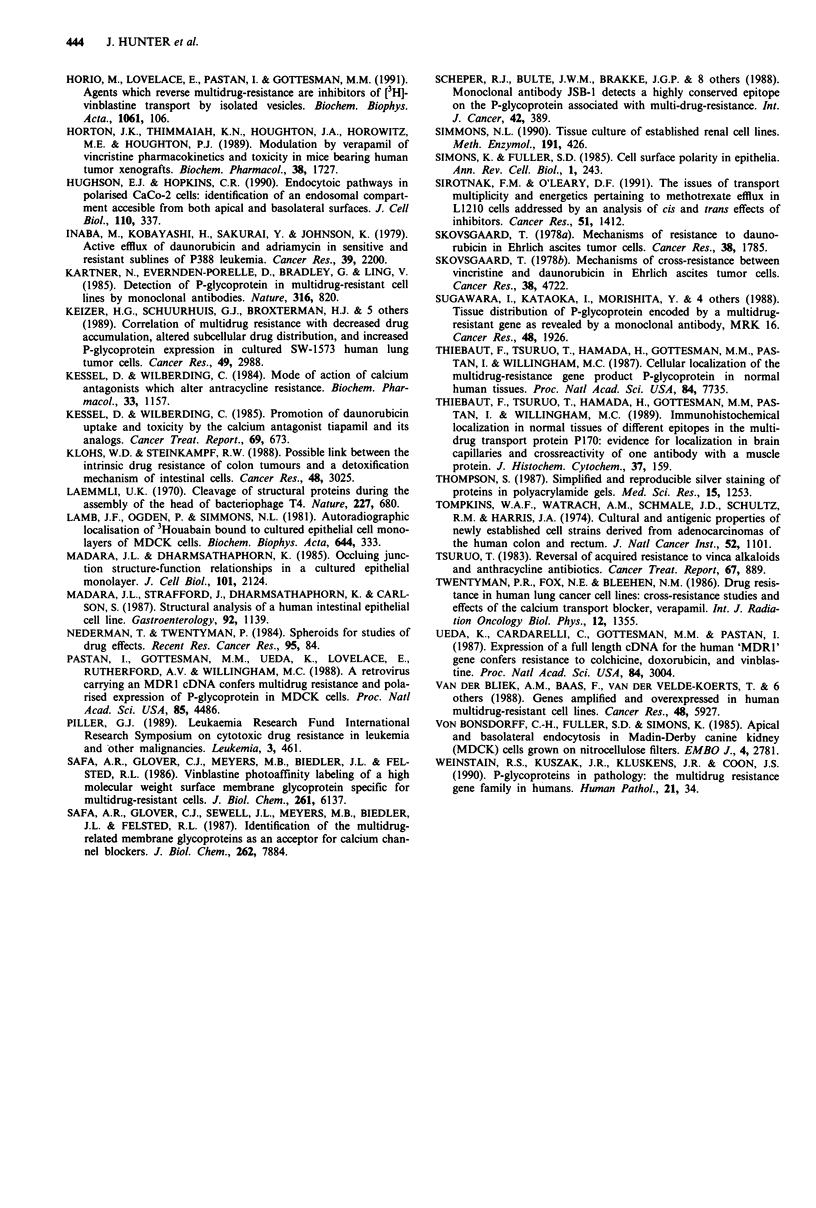# Epithelial secretion of vinblastine by human intestinal adenocarcinoma cell (HCT-8 and T84) layers expressing P-glycoprotein.

**DOI:** 10.1038/bjc.1991.328

**Published:** 1991-09

**Authors:** J. Hunter, B. H. Hirst, N. L. Simmons

**Affiliations:** Gastrointestinal Drug Delivery Research Centre, University of Newcastle upon Tyne, Medical School, UK.

## Abstract

**Images:**


Br. J. Cancer (1991), 64, 437–444 © Macmillan Press Ltd., 1991

### Epithelial secretion of vinblastine by human intestinal adenocarcinoma cell (HCT-8 and T84) layers expressing P-glycoprotein

J. Hunter, B.H. Hirst & N.L. Simmons

*Gastrointestinal Drug Delivery Research Centre and Department of Physiological Sciences, University of Newcastle upon Tyne, Medical School, Newcastle upon Tyne NE2 4HH, UK.*

**Summary** P-glycoprotein expression was demonstrated in two human intestinal adenocarcinoma cell-lines (HCT-8, ileocaecal and T84, colonic) by immunoprecipitation of a 170–180 kDa protein with monoclonal antibody JSB-1. Both HCT-8 and T84 formed functional epithelial cell layers of high transepithelial electrical resistance (>700 Ω ·cm$^2$) when grown on permeable matrices. These epithelial layers demonstrated vectorial secretion (net vinblastine fluxes in the basal-to-apical direction of 0.135 and 0.452 pmol h$^{-1}$ cm$^{-2}$ in HCT-8 and T84 cell layers, respectively, from bathing solutions containing 10 nM vinblastine). These vectorial vinblastine secretions were sensitive to inhibition by verapamil. Passive transepithelial vinblastine permeation was limited by the presence of intercellular (tight) junctions, as demonstrated by the high transepithelial electrical resistance, and verapamil increased this passive vinblastine permeation concomitant with a reduction in the electrical resistance. Cellular vinblastine loading was significantly greater from the basal side, and this was also susceptible to inhibition by basal verapamil. The demonstration of vectorial transport of vinblastine in human intestinal colonic adenocarcinoma cell layers is direct evidence in favour of the hypothesis that the function of mdr1 in epithelia from the gastrointestinal tract is to promote detoxification by a process of epithelial secretion. This study also highlights that cellular vinblastine accumulation depends not only upon P-glycoprotein function, but also upon differential apparent membrane permeabilities and the presence of intercellular (tight) junctions that may restrict drug permeation and cellular accumulation to apical or basal membrane domains.

The insensitivity of solid neoplasms to conventional chemotherapy has justly received considerable attention. It is increasingly apparent that certain intrinsic cellular properties of these tumour cells, often shared by the tissue of origin, contribute to such insensitivity. In a survey of 20 human colon carcinoma cell-lines (Chantret *et al.*, 1988) the greater majority were shown to possess the basic elements of epithelial structure (cellular polarity including the formation of distinct membrane domains, and formation of tight junctions between cells) and even function (formation of a barrier by restriction of free diffusion, and fluid filled blisters resulting from transepithelial salt and water absorption). In two cases the expression of histotypic differentiation (apical brush-border, and brush-border specific hydrolases) was so extreme, as to allow the use of such tumour cells as valid *in vitro* models of the human intestinal epithelium.

P-glycoprotein is a 170–180 kDa membrane glycoprotein associated with the phenomenon of pleiotropic (multidrug) resistance (MDR). Immunohistochemical techniques have demonstrated the presence of P-glycoprotein in the apical membranes of several epithelia (Sugawara *et al.*, 1988; Cordon-Cardo *et al.*, 1990; Thiebaut *et al.*, 1989; Thiebaut *et al.*, 1987), especially in the gastrointestinal tract, e.g., jejunum, colon, liver (bile duct canaliculi), and pancreas, indicating some intrinsic function (Cordon-Cardo *et al.*, 1990). Retroviral transfection of a dog kidney epithelial cell-line (MDCK) with mdr1 cDNA results in polarised expression of P-glycoprotein to the apical plasma membrane domain (Pastan *et al.*, 1988).

In many cases, tumours developing from epithelial tissues tend to possess an innate resistance to the anticancer drugs prior to any treatment regime being performed (Fojo *et al.*, 1987; Klohs & Steinkampf, 1988; Goldstein *et al.*, 1989). Thus it has been hypothesised that the P-glycoprotein is involved in detoxification mechanisms, along with other enzymic systems such as glutathione-S-transferases and cytochrome P-450 (Deffie *et al.*, 1988; Piller 1989), pumping ingested toxins out of the cells into the lumen of the gastrointestinal tract. A vectorial transport of substrate (vinblastine) in *mdr*1 transfected MDCK cells has indeed been demonstrated (Horio *et al.*, 1989).

The exact mechanism by which the function of the 170–180 kDa glycoprotein mediates pleiotropic drug resistance is not fully understood, but it is now broadly accepted that reduced intracellular drug accumulation resulting from increased active (ATP-dependent) drug efflux is an important factor in the mechanism of resistance (Dano, 1973; Skovsgaard, 1978*a*; Skovsgaard, 1978*b*; Inaba *et al.*, 1979). Vinblastine (and verapamil, see below) specifically bind to the 170–180 kDa glycoprotein (Cornwell *et al.*, 1986; Cornwell *et al.*, 1987; Safa *et al.*, 1987), suggesting a functional correlation between the levels of the membrane glycoprotein in multidrug resistant cells and the level of binding of a specific chemotherapeutic agent (Saga *et al.*, 1986). The levels of expression of the 170–180 kDa glycoprotein correlate with the degree of drug resistance (Ueda *et al.*, 1987; Van der Bliek *et al.*, 1988; Keizer *et al.*, 1989). Calcium antagonists (e.g., verapamil), calmodulin inhibitors (e.g., trifluoperazine) and other agents (e.g., reserpine) inhibit the active drug efflux and restore drug sensitivity in multidrug resistant cells (Tsuruo, 1983; Kessel & Wilberding, 1984; Kessel & Wilberding, 1985; Bellamy *et al.*, 1988; Twentyman *et al.*, 1986). Although the precise mechanism of these agents is not yet elucidated, their use provides a convenient functional definition for P-glycoprotein-mediated fluxes in intact cells and tissues.
test directly the hypothesis that expression of P-glycoprotein is associated with transepithelial secretion (the generation of a net flux of substrate in a basal-to-apical direction) of MDR-substrates such as vinblastine sulphate in epithelial tumour cells arising from the gastrointestinal tract. Secondly, to determine whether the intrinsic expression of P-glycoprotein in a functional context within a polarised epithelium places constraints upon attempts to modify or circumvent P-glycoprotein function, such as with the use of inhibitors.

In this study we have chosen two human adenocarcinoma cell-lines, HCT-8 (human ileocaecal adenocarcinoma) and T84 (human colonic adenocarcinoma). Both cell-lines demonstrate considerable intestinal-like differentiation (Dharmsathaphorn *et al.*, 1984; Allen *et al.*, 1991) and are capable of forming epithelial monolayers when grown upon permeable

Received 30 November 1990; and in revised form 1 May 1991.

matrices, so allowing the *in vitro* characterisation of transepithelial solute fluxes concomitant with their biophysical parameters (Madara *et al.*, 1987; Chan *et al.*, 1989).

### Materials and methods

### Cell culture

HCT-8 and T84 cells were obtained from the ATCC (Rockville, Maryland 20852 USA). HCT-8 cells were maintained in RPMI 1640 with 10% horse serum, 1 mM glutamine, 1 mM sodium pyruvate, 40 IU $ml^{-1}$ penicillin and 40 $\mu g\ ml^{-1}$ streptomycin. T84 cells were grown in a 1:1 mixture of Dulbecco's modified Eagle's medium and Ham's F-12 with 5% newborn calf serum and 200 IU $ml^{-1}$ penicillin, 200 $\mu g\ ml^{-1}$ streptomycin. It should be noted that the cell lines used in this present study have not been selected for drug resistance (Tompkins *et al.*, 1974; Dharmsathaphorn *et al.*, 1984). Confluent monolayers were subcultured every 5–7 (HCT-8) days or 14–16 days (T84), by treatment with 0.05% trypsin and 0.02% EDTA in $Ca^{2+}$-and $Mg^{2+}$-free phosphate-buffered saline (PBS). The cultures were incubated at 37°C in a humidified atmosphere of 5% $CO_2$/95% air.

For uptake/efflux studies HCT-8 cells were seeded into 24-well Linbro plates (Costar) at a density of $4 \times 10^5$ cells/well, and grown for 5 days under the above conditions, until confluent monolayers were produced.

Functional epithelial layers of HCT-8 and T84 cells on permeable supports were prepared essentially as described by Dharmsathaphorn *et al.* (1984) and Simmons (1990). Cells were seeded at high density ($1 \times 10^6$ cells $ml^{-1}$, 3 ml well) into rat-tail collagen coated filtercups, Transwell 24.5 mm diam. (Costar) for HCT-8, or Anocell 25 mm diam. (Anotec) for T84 cells. Filtercups were then cultured in 6-well plates at 37°C, 5% $CO_2$ for 1 week or 1 month for HCT-8 and T84, respectively, with medium replacement every 2–3 days. The formation of functional epithelial layers was monitored by the development of a significant transepithelial resistance ($R_t$), as measured using a WPI Evometer fitted with 'chopstick' electrodes to allow transepithelial current passage and potential sensing (Simmons, 1990). Cell monolayers were used when the transepithelial resistance typically exceeded 600 or 900 $\Omega \cdot cm^2$ for the HCT-8 and T84 cells, respectively (values of resistance for filter cups, ~300 $\Omega \cdot cm^2$ are subtracted from all data).

### Uptake/efflux of $^3$H-vinblastine in HCT-8 cells

The growth medium was removed and the cell layers washed rapidly by plunging into 5 l PBS and the Linbro plates blotted dry. Serum-free medium, 1 ml at room temperatue, containing 10 nM [$^3$H]-vinblastine sulphate, in the presence or absence of 0.2 mM verapamil, was then added to each well. Incubations for various times were performed at 37°C. [$^3$H]-Vinblastine uptake was terminated by removal of isotope-containing medium, the plates were washed as described above, and 0.5 ml trypsin/EDTA (0.05%/0.2%) solution added. The resulting cell suspensions were collected into scintillation vials. $^3$H-Activities (d.p.m.) were determined by liquid scintillation counting (Beckman LS 5000CE spectrometer) using LKB 'Optiphase safe' scintillation cocktail. Appropriate corrections were made for quenching effects.

A second group of cell layers were preloaded for 4 h with 10 nM $^3$Hvinblastine sulphate in 1 ml serum-free medium in each well. Isotope-containing medium was then discarded and the cell layers washed, as described above. Fresh serum-free medium (1 ml) was then added to each well, either in the absence or presence of 0.2 mM verapamil, and the cells incubated for various times at 37°C. Following incubation the amount of $^3$Hvinblastine contained within the cell layers was determined exactly as described for the uptake measurements.

Uptake/efflux of $^3$Hvinblastine is expressed relative to cell contents at 4 h loading with 10 nM $^3$Hvinblastine; each incubation condition was performed in quadruplicate.

### Measurement of bidirectional transepithelial [$^3$H]-vinblastine sulphate fluxes

Measurements of transepithelial solute flux were made essentially as described by Simmons (1990). Functional epithelial layers in filter cups were washed with 2 × 3 ml serum-free medium and placed into fresh 6-well plates containing 3 ml serum-free medium (basal solution), a further 3 ml serum-free medium was then pipetted into the upper chamber (apical solution) of the filter cup. Transepithelial resistance was measured following 10 min incubation of the cells at 37°C, as described above.

The medium on either the apical, or basal side of the monolayers was then removed and replaced with 3 ml serum-free medium containing 10 nM [$^3$H]-vinblastine sulphate in the apical (a) or basal (b) solutions, in the presence or absence of 0.2 mM verapamil, followed by incubation at 37°C. The concentration of vinblastine was chosen to be considerably below the $K_m$ for vinblastine transport (Horio *et al.*, 1991) and therefore well within the first-order part of the saturation curve, while the concentration of verapamil was chosen to completely block P-glycoprotein function. In order to measure the bidirectional fluxes of vinblastine sulphate ($J_{a-b}$, flux from apical to basal solutions, and $J_{b-a}$, flux from basal to apical solutions), 100 µl samples of medium from each side of the monolayer were taken at regular intervals, $^3$H activities in these samples were then measured as described above. Each incubation was performed at least in triplicate. On completion of the experiments, the filter cups were washed by immersion in 1 l PBS, the filters removed from their holders and placed into scintillation vials and $^3$H activities determined.

Inulin is a high molecular weight (~5,000) inert macromolecule which is normally excluded from cells, and is therefore used as a tracer of extracellular permeability pathways through the intercellular tight junctions, and an indicator of changes in the permeability of this paracellular pathway (Madara & Dharmsathaphorn, 1985). Bidirectional [$^{14}$C]-inulin fluxes were determined exactly as for vinblastine sulphate fluxes.

### Assessment of mitochondrial enzyme function: MTT assay

Cells were seeded onto 96-well plates (10,000 cells/well) and the monolayers were allowed to grow over the following 48 h. The cells were washed with 100 µl PBS before addition of verapamil (0–500 µM) or vinblastine (0–200 nM). Stock solutions of verapamil were freshly prepared in dimethyl sulphoxide at a concentration of 10 mg $ml^{-1}$, followed by dilution in serum-free medium. Control wells were included in each plate (each solution contained the same concentration of DMSO). Cells were then incubated for either 1 or 5 h under cell culture conditions. On completion of the incubation, 50 µl of MTT reagent (consisting of 5 mg 3-[4,5-dimethylthiazo-2-yl]-2,5-diphenyltetrazolium bromide and 1.5 mg phenazine methosulphate per 5 ml PBS) was added to each well. The plates were then incubated for a further 20 min at 37°C. The cell monolayers were fixed by the further addition of 100 µl of buffered formalin, pH 7.0, to each well for 1 h. After overnight drying in air, the formazan product was extracted by addition of 100 µl acidified isopropanol to each well (1 ml 1 M HCl acid per 100 ml isopropanol) before absorbance at 570 nm was measured with a Dynatech MR700 ELISA plate reader (Allen *et al.*, 1991).

### Assessment of lysosomal integrity: neutral red assay

Cells were plated onto 96-well plates and incubated with verapamil as described above, following incubation the solutions were removed and the cells washed with 100 µl PBS/well. The cells were then incubated for 3 h with 200 µl neutral red (50 µg $ml^{-1}$ freshly prepared in serum-free medium). The monolayers were fixed by the addition of 100 µl 1% formalin containing 1% $CaCl_2$ for 2–3 min. This solution was then discarded and the plates were allowed to dry in air before the dye was extracted with a solution containing 1%

acetic acid, 50% ethanol (100 μl per well) over a period of 45 min with shaking. Absorbance was measured at 540 nm with an ELISA plate reader (Allen *et al.*, 1991).

### *Immunoprecipitation of P-glycoprotein*

Immunoprecipitates of the P-glycoprotein were prepared essentially as described by Hamada *et al.* (1987). Briefly, $5 \times 10^7$ cells were detached by scraping, washed in ice cold 0.15 M NaCl, 0.02 M sodium phosphate buffer, pH 7.4, and centrifuged at 1,000*g* to form a cell pellet. The cell pellets were resuspended in 10 ml buffer S (50 mM Tris/HCl (pH 8), 140 mM NaCl, 5 mM NaF, 2 mM sodium vanadate, 0.2 mM PMSF, 0.25 μg ml$^{-1}$ aprotinin, 4 mM EDTA, 0.5% sodium deoxycholate) and incubated on ice for 30 min. The suspensions were clarified by centrifugation at 10,000*g* for 20 min. An aliquot, 4 ml, of this cell extract was then incubated with 100 μl monoclonal antibody to P-glycoprotein, JSB-1 (Scheper *et al.*, 1988), with shaking for 2 h at 4°C. Protein A-Sepharose CL-4B, 200 μl, was then added (25% v/v in buffer S) and incubated for a further 30 min at 4°C with shaking. A pellet was formed by centrifugation (400 *g* for 2 min), and this pellet was washed five times with 2 ml ice cold buffer S before 150 μl sample buffer (Laemmli, 1970) was added and the precipitates analysed by SDS-polyacrylamide gel electrophoresis using 5–15% linear gradient gels (Laemmli, 1970). Gels were fixed and stained using a method as described by Thompson (1987).

### *Materials*

[$^3$H]-vinblastine sulphate and [$^{14}$C]-inulin were purchased from Amersham International plc (Little Chalfont, Amersham, Bucks). The scintillation cocktain 'Optiphase safe' and Protein-A Sepharose CL-4B were obtained from Phamacia LKB Biotechnology Ltd (Milton Keynes, Bucks). All tissue culture media and reagents (Gibco BRL) and tissue culture plastics (Nunc) were supplied by Life Technologies Ltd (Paisley, Scotland). The filter cups used for this study were as follows: for HCT-8 cells Transwells for 6-well plates, with 0.4 μm pore size and 24.5 mm diam (Costar Nucleopore UK Ltd, High Wycombe, Bucks); for T84 cells, Anocell 25 tissue culture inerts, with 0.2 μm pore size and 25 mm diam (Anotec Separations Ltd, Oxon). The monoclonal antibody JSB-1 was purchased from Sera-lab Ltd (Crawley Down, Sussex), all other chemicals were obtained from Sigma Chemical Co (Poole, Dorset) or BDH Chemicals Ltd (Poole, Dorset).

### **Results**

### *Cellular vinblastine uptake and efflux in HCT-8 cells*

With HCT-8 cells grown as monolayers on plastic plates, cellular uptake and loss of vinblastine sulphate (Figure 1) will represent the relative rates of influx and efflux mainly across the apical cell border. Vinblastine accumulation in HCT-8 cells appeared bi-phasic and did not approximate to first-order kinetics. Verapamil increased the extent of cellular uptake of vinblastine by 38% at 5 h (Student's *t*-test, $P < 0.05$; Figure 1a). Vinblastine loss from HCT-8 cell monolayers occurred only after a delay of approximately 60 min (Figure 1b). Verapamil inhibited vinblastine loss from preloaded cells by 38% at 3 h (Student's *t*-test, $P < 0.05$; Figure 1b). The time-dependent increase in accumulation of vinblastine and the inhibition of vinblastine loss by verapamil are thus consistent, i.e., the expression of a verapamil-sensitive efflux of vinblastine across the apical cell border. Similar experiments with T84 cells were not possible due to the detachment of cells with washing.

### *Epithelial properties of filter-grown cell layers*

HCT-8 cells when grown to confluency in permeable filter cups developed a transepithelial electrical resistance of

**Figure 1** Cellular **a**, uptake and **b**, efflux of [$^3$H]-vinblastine sulphate in HCT-8 cells grown on plastic. Results are illustrated as the mean, with error bars of 1 s.e. (where error bars are not seen 1 s.e. ≤ size of symbols used), vinblastine content expressed as a percentage of the total cell content, where 100% = total amount of [$^3$H]-vinblastine sulphate in cells after a 4 h incubation with drug, for four observations on the same plate. Vinblastine uptake was not significantly different in the absence or presence of verapamil ($P = 0.105$), while vinblastine efflux was significantly greater in the presence of verapamil ($P < 0.001$), as analysed by two-way analysis of variance. Results are illustrative of 4–5 separate experiments.

746.5 ± 47.9 Ω·cm$^2$ ($n = 70$) after 7 days in culture. T84 cells reached a transepithelial electrical resistance of 1028.9 ± 36.1 Ω·cm$^2$ ($n = 25$) after 28–35 days of growth. These values are characteristic of 'tight' epithelia such as the colon. HCT-8 and T84 cells display a small potential differences ($V_t$), basal electropositive, of 1.0 (HCT-8) or 0.4 mV (T84) when grown under these conditions (Chan *et al.*, 1989) indicating the development of functional ion ($Na^+$, $Cl^-$) transepithelial transport.

### *Transepithelial vinblastine flux*

Using these functional HCT-8 and T84 epithelial layers, time-dependent movement of vinblastine sulphate was measurable, both in the apical-to-basal and basal-to-apical directions (Figure 2). The basal-to-apical flux ($J_{b\text{-}a}$) was 0.234 ± 0.007 ($n = 6$) and 0.547 ± 0.012 ($n = 12$) pmol h$^{-1}$ cm$^{-2}$, and this exceeded the apical-to-basal flux ($J_{a\text{-}b}$) of 0.099 ± 0.008 ($n = 7$) and 0.095 ± 0.017 ($n = 4$) pmol h$^{-1}$ cm$^{-2}$, in the HCT-8 and T84 epithelial layers, respectively. A net flux ($J_{net} = J_{b\text{-}a} - J_{a\text{-}b}$) was, therefore, observed in the basal-to-apical direction for both cell lines. The magnitude of the directional net flux being 0.135 and 0.452 pmol h$^{-1}$ cm$^{-2}$ for HCT-8 and T84, respectively, which is 1.4- or 5.75-fold greater than the unidirectional flux in the apical-to-basal direction for these cell layers. The net flux cannot be accounted for by the small electrical potential differences maintained by the epithelial layers in the experimental conditions used.

Transepithelial electrical resistance was not compromised over the time course of the experiments. A slight increase in $R_t$ was observed in both cell lines as they became equilibrated to the fresh medium; from 746.5 ± 47.9 ($n = 70$) to 839.3 ±

**Figure 2** Transepithelial vinblastine sulphate fluxes measured in **a**, HCT-8 and **b**, T84 cells. Mean, with error bars of 1 s.e., $^3$Hvinblastine flux was measured in the basolateral-to-apical (B-A: HCT-8, $n = 6$; T84, $n = 12$), apical-to-basolateral (A-B: HCT-8, $n = 7$; T84, $n = 4$), and in the basolateral-to-apical direction in the presence of 0.2 mM verapamil (B-A verapamil: HCT-8, $n = 16$; T84, $n = 5$). The line illustrates the least squares best fit to the data, the slopes of which give the vinblastine flux. Data are from five different experiments.

**Figure 3** Scatter diagram of apparent permeabilities of vinblastine sulphate in HCT-8 (●) and T84 (▲) epithelial layers plotted against transepithelial electrical resistance. The apparent permeability ($J_{a\text{-}b}/C$), calculated as the flux of vinblastine $J_{a\text{-}b} \div$ vinblastine concentration, has units of cm h$^{-1}$.

46.1 Ω·cm$^2$ ($n = 42$), and from 1028.0 ± 36.1 ($n = 25$) to 1241 ± 52.6 Ω·cm$^2$ ($n = 12$) in the HCT-8 and T84 cell layers, respectively. Thus, vinblastine sulphate, at the concentrations used, did not materially alter the barrier formed by the HCT-8 and T84 cells.

The integrity of this barrier in HCT-8 cells was also evaluated using [$^{14}$C]-inulin flux, in parallel with the vinblastine sulphate measurements. After 200 min using a concentration of 1 μM [$^{14}$C]-inulin, the apparent permeability (J/C; i.e., inulin flux per cm$^2$ ÷ initial concentration of inulin) was $2.74 \times 10^{-4}$ ($J_{a\text{-}b}$) and $1.82 \times 10^{-4}$ ($J_{b\text{-}a}$) cm h$^{-1}$; that is an order of magnitude lower than that seen for vinblastine flux (see below and Figure 3).

Verapamil, at a concentration of 0.2 mM, reduced the net transport of vinblastine sulphate across the epithelial cell layers, primarily by an approximate 50% reduction in basal-to-apical flux in both cell lines (Figure 2). This effect was similar with verapamil added to either the apical or basolateral solutions; e.g. in HCT-8 cell layers, $J_{a\text{-}b}$ was 0.102 ± 0.006 ($n = 8$) and 0.126 ± 0.001 ($n = 6$) with basal and apical verapamil, respectively, giving an average flux of 0.110 ± 0.004 ($n = 14$) pmol h$^{-1}$ cm$^{-2}$ illustrated in Figure 2a.

In the presence of verapamil, 0.2 mM, for 300 min the transepithelial electrical resistance fell from control values of 839.3 ± 46.1 Ω·cm$^2$ ($n = 42$) to 656.8 ± 68.2 Ω·cm$^2$ ($n = 9$) in the HCT-8 cell layers, a decrease of 21.7%. In the T84 cell layers, a 73.9% decrease in $R_t$ was observed, from 1241.1 ± 52.6 ($n = 12$) to 323.5 ± 62.6 Ω·cm$^2$ ($n = 6$). The verapamil-dependent decrease in electrical resistance correlates with the extent of drug leak across the T84 epithelial layers, reducing the apparent inhibitory effect of verapamil on the basal-to-apical flux (Figure 2b).

Figure 3 illustrates a scatter diagram of the apparent permeability of vinblastine sulphate (from individual data for $J_{a\text{-}b}$) in T84 and HCT-8 epithelial layers plotted against the transepithelial electrical resistance ($R_t$). $R_t$ is a measure of the 'tightness' of the epithelium (i.e. the extent of the non-cellular (paracellular or junctional) pathway for solute permeation). For T84 and HCT-8 epithelia it is clear that the unidirectional apparent permeability for apical-to-basal flux ($J_{a\text{-}b}/C$) is inversely related to $R_t$. This relationship is entirely consistent with a parallel permeation pathway model for vinblastine sulphate, in which a cellular (hydrophobic) rate at limiting resistance values (< 600 kΩ·cm$^2$) is present together with a paracellular (hydrophilic) rate (see Figure 5). The decrease in electrical resistance seen with verapamil in T84 epithelial layers is thus correlated with increased 'leakage' of vinblastine sulphate via a paracellular route.

*Cell-associated vinblastine content*

The levels of cell-associated vinblastine sulphate after 200 min incubation was dependent on several factors, including the side of the cell layer exposed to the drug. A significantly increased vinblastine level was measured in those cell layers exposed to the drug from the basolateral surface compared to the apical surface (Table I). In the HCT-8 cells cellular vinblastine content was 1.8-fold greater when loading was from the basal side. In the T84 cells, the cellular vinblastine content was approximately 5-fold greater with basal loading. These differences cannot be explained by tracer binding to the filter matrix on which the cells are grown.

In the HCT-8 cell layers no difference was seen in cellular vinblastine content as a consequence of apical or basal exposure until the transepithelial electrical resistance of the layers exceeded ~300 Ω·cm$^2$. For example, when HCT-8 cell layers with an average resistance of 298.5 ± 32.1 Ω·cm$^2$ ($n = 4$) were loaded with vinblastine sulphate, as above, the cell associated drug was found to be either 0.111 pmol cm$^{-2}$ ($n = 2$) (drug presented apically) or 0.107 pmol cm$^{-2}$ ($n = 2$) (drug presented basally); i.e. no difference. This implies that access of vinblastine sulphate to either epithelial surface occurs, whether label is present in apical or basal solutions in low resistance (leaky or incomplete) layers.

**Table I** Levels of cell-associated vinblastine sulphate

| | | *Cell-associated vinblastine (pmol cm$^{-2}$)* | |
|---|---|---|---|
| *Cell line* | *Verapamil* | *Apical loading* | *Basal loading* |
| HCT-8 | – | 0.147 ± 0.006 ($n = 13$) | 0.269 ± 0.029 ($n = 12$) |
| HCT-8 | Basal | 0.174 ± 0.011 ($n = 3$) | 0.183 ± 0.011 ($n = 3$) |
| HCT-8 | Apical | 0.136 ± 0.012 ($n = 3$) | 0.328 ± 0.025 ($n = 3$) |
| T84 | – | 0.260 ± 0.042 ($n = 3$) | 1.310 ± 0.154 ($n = 6$) |

Values are expressed as mean ± 1 s.e. cell-associated vinblastine content after 5 h incubation with 10 nM vinblastine. Verapamil, 0.2 mM was added to basal or apical surface as indicated.

Verapamil was found to decrease the vinblastine content of the cells by approximately 14%, when presented together with the radioligand on the basolateral surface of the cell layer (Table I). In contrast, a 29% increase in cell vinblastine content was observed when verapamil was presented on the apical surface, with [$^3$H]-vinblastine on the basolateral surface (Table I). No significant difference in the lower levels of cellular vinblastine loading with apical presentation was evident as a result of verapamil treatment (Table I).

### *Assessment of acute verapamil and vinblastine toxicity*

A high concentration of verapamil was employed in these experiments to inhibit P-glycoprotein function. In preliminary experiments in HCT-8 and MDCK cells, verapamil, 200 µM, was required to reduce the basal-to-apical flux of vinblastine to that of the passive apical-to-basal flux rate, i.e. 100% inhibition (e.g., see Figure 2a). HCT-8 cells showed no reduction in viability upon incubation for 5 h, the maximum duration of the flux experiments, with concentrations of verapamil upto and including 200 µM (Figure 4a). However, at higher concentrations, above 300 µM, the reduction of formazan dye was increased, consistent with an increased cellular permeability to the MTT reagent, with no evidence of reduction in mitochondrial enzyme function. Acute incubation with vinblastine, 0.1–200 nM, for 5 h did not effect HCT-8 cell viability assessed by the MTT assay. Similar exposure of the cells to vinblastine followed by removal of the vinblastine, and culture for a further 48 h, is, however, associated with a reduction of formazan dye production, consistent with the well recognised cytostatic action of this drug (J. Hunter, C.N. Allen, N.L. Simmons & B.H. Hirst, unpublished data).

Using the neutral red assay to assess toxicity (Figure 4b), a relatively low concentration of verapamil, 20 µM, was associated with an approximate 20% reduction in lysosomal and/or plasma membrane permeability, but this was not reduced further by increasing concentrations of verapamil.

### *Immunoprecipitation of P-glycoprotein*

Immunoprecipitation of whole cell extracts (same cell number) of HCT-8 and T84 with the monoclonal antibody JSB-1 followed by SDS-PAGE analysis yielded the protein band patterns seen in Figure 5. A single diffuse band was detected by silver staining, with a $M_r$ value of 170–180 kDa consistent with expression of P-glycoprotein in both cell types. It is also clear from the gel that a greater amount of the protein is present within T84 cells when compared to HCT-8 cells. No high $M_r$ bands were visible on gels if either the JSB-1 antibody or the cell extracts were omitted.

**Figure 4** Changes in **a**, mitochondrial enzyme activity (MTT) and **b**, neutral red uptake in HCT-8 cells after incubation with various concentrations of verapamil for 5 h. Results are illustrated as the mean, with error bars of 1 s.e. ($n$ = 8), MTT activity or neutral red uptake expressed as a percent of control activities in the absence of verapamil.

**Figure 5** Demonstration of P-glycoprotein in HCT-8 and T84 cells. Silver-stained SDS-polyacrylamide gels of immunoprecipitates (with monoclonal antibody JSB-1) of cell extracts of HCT-8 or T84 cells. $M_r$ values indicate the position of Sigma high MW markers (kDa), while the arrow indicates a $M_r$ of 170 kDa.

### Discussion

In polarised monolayers of epithelial cells formed by two separate human intestinal adenocarcinomas cell-lines (T84 and HCT-8), we have demonstrated net secretion of vinblastine sulphate from basal to apical cell surfaces. A model illustrating the features of the net flux is presented in Figure 6. There are several lines of evidence to suggest that this net secretory flux is the functional result of P-glycoprotein expression in T84 and HCT-8 cells. Firstly, immunoprecipitation of protein from both cell types of monoclonal antibody JSB-1 results in a protein that has a molecular mass entirely consistent with the *mdr*1 gene product (Figure 5). Secondly, in cells grown upon plastic substrate, a component of cellular efflux of vinblastine sulphate is inhibited by verapamil, whilst loading of cells by vinblastine is increased by verapamil (Figure 1). Thirdly, net secretory flux is itself inhibited by verapamil (Figure 2). Finally, the increased amount of P-glycoprotein expression detected in T84 cells (Figure 5) correlates with the greater vinblastine flux asymmetry in this cell-line (Figure 2). In a study of vectorial transport of vinblastine by dog kidney cells transfected with a retroviral vector containing *mdr*1, increased vectorial transfer was seen upon transfection (Horio *et al.*, 1989). The present data are, therefore, the first direct demonstration in favour of the hypothesis that the function of *mdr*1 in epithelia from the gastrointestinal tract is to promote detoxification by a process of epithelial secretion.

Determination of expression of P-glycoprotein by immunoprecipitation relies on the use of monoclonal antibody JSB-1 which recognises a highly conserved epitope, most probably on a cytoplasmic domain of the protein (Scheper *et al.*, 1988). Monoclonal antibody C219 also recognises a cytoplasmic epitope of the protein (Kartner *et al.*, 1985). In our hands antibody C219 weakly immunoprecipitates a band of circa 170 kDa, but in addition a number of other major bands are present in the immunoprecipitate suggesting that the epitope recognised by C219 is not unique to P-glycoprotein, even in non-muscle cells (Thiebaut *et al.*, 1989). Previous studies on HCT-8 cells have shown that although verapamil increases adriamycin accumulation and cytotoxicity, surface labelling failed to detect a glycoprotein of appropriate molecular size due to the insensitivity of the technique (Klohs & Steinkampf, 1988). The exact cellular location of P-glycoprotein in HCT-8 and T84 cells has not been determined, but the presence of vectorial transepithelial transport of vinblastine is consistent with a polarised expression to the apical cell surface. It should be noted that Broxterman *et al.* (1989) localised a significant portion of cellular immunofluorescence in drug resistant cells to intracellular vesicular structures. Active drug accumulation into an endomembrane (acidic) vesicular compartment, with subsequent exocytotic release at the cell surface has been implicated in P-glycoprotein mediated drug efflux (Beck, 1987). The non-first order kinetic behaviour of vinblastine accumulation and efflux in HCT-8 cells (Figure 1) would be consistent with such a mechanism. Vesicular accumulation of vinblastine would not preclude vectorial transfer across the epithelal monolayer, provided that such vesicular traffic was polarised with final delivery to the apical membrane (Figure 6). Polarisation in endocytotic trafficking mechanisms from the apical and basolateral surfaces has been demonstrated in Caco-2 colonic adenocarcinoma cells (Hughson & Hopkins, 1990). It has also been reported that a significant transcytosis (bulk directional transcellular transport by a population of endocytotic vesicles) of extracellular marker compounds exists in Caco-2 cells, oriented from basal to apical cell surfaces (Heyman *et al.*, 1990). Could such a mechansim account for the vectorial transfer of vinblastine reported here? Direct determination of bidirectional inulin fluxes showed that these are at least an order of magnitude less than those of vinblastine, and non-vectorial in nature, demonstrating that an active accumulation must exist even with a vesicular model for transepithelial vinblastine permeation.

**Figure 6** Model for vinblastine secretion across intestinal adenocarcinoma epithelial cell layers. The passive permeability of the apical and basolateral membranes, and junctional complexes (tight junctions; tj) are illustrated, which together with active apical vinblastine efflux mediated via P-glycoprotein (●), give rise to the bidirectional fluxes ($J_{a\text{-}b} < J_{b\text{-}a}$) resulting in a net flux ($J_{net}$) in the basal-to-apical direction. Possible vectorial transport via a vesicular compartment is illustrated. The diagram also illustrates the three sites (**a,b,c**) of action of verapamil ($V_p$) idenified in the present study.

The present data lend support to the linkage between the intrinsic drug resistance of colon tumours and a detoxification mechanism present within normal cells (Klohs & Steinkampf, 1988; Horton *et al.*, 1989). Additionally, the demonstration of net vinblastine transport across an epithelial layer in which the basal bathing solution is progressively depleted of vinblastine suggests that an epithelial surface formed within a solid tissue mass or multicellular spheroid could generate a compartment that was relatively depleted of cytotoxic drug (Nederman & Twentyman, 1984). Thus, cells displaying epithelial junction formation and P-glycoprotein expression could confer 'resistance' to an enclosed non-differentiated cell population. Little direct experimental data is available at present to support this contention, but we note that epithelial 'spheroids' composed of an outer differentiated cell-layer enclosing a progressively de-differentiated cell mass are considered, by some, as more realistic models of solid tumour cell systems (Nederman & Twentyman, 1984). In colonic tumours *in situ*, P-glycoprotein expression was inhomogeneous in the tumour, but was enhanced at the luminal membrane face (Cordon-Cardo *et al.*, 1990); in situations where a tumour mass and invasion front into underlying tissue could be defined, P-glycoprotein has been reported to be preferentially localised in cells at the invasion front (Weinstein *et al.*, 1990).

The reconstitution *in vitro* of intestinal epithelial function in monolayer culture in filter cups allows experimental access to both relevant cell surfaces and provides a convenient system to test cytotoxic regimes. Of special interest in this context are polar compounds that may be subject to significant diffusion restriction by the formation of a 'tight' epithelial barrier (Madara *et al.*, 1987). Thus inulin possesses an extremely low permeability in comparison to vinblastine, whose transepithelial permeability is likely to be predominantly transcellular in such highly resistive layers. It should be noted that as epithelial resistance decreases, apical-to-basal vinblastine permeability increases, suggesting that significant diffusion via a non-cellular polar route (the paracellular route) occurs (see Figure 3). This result suggests that if significant diffusion restriction occurs by tight junction formation within a solid tumour mass, enhanced drug permeation could be achieved by agents or drugs disrupting epithelial junction integrity (Figure 6). Of interest in this context is the marked reduction in transepithelial resistance of T84 epithelial layers upon incubation with verapamil that is coincident with enhanced apical-to-basal 'leak' flux of vinblastine.

An additional facet of the expression of an epithelial phenotype is the existence of separate apical and basolateral plasma membrane domains with a unique lipid and integral membrane protein composition (Simons & Fuller, 1985). Also membrane amplification (via the presence of a brush-border or extensive interdigitations of the basolateral surface) can significantly alter the effective surface areas for drug diffusion into the cytosol. In the extensively studied MDCK dog kidney cell-line, careful morphometric data show that the basolateral surface area exceeds that of the apical membrane by 7–10-fold (von Bonsdorff *et al.*, 1985; Lamb *et al.*, 1981). For epithelial colonic adenocarcinoma cells grown upon plastic the basolateral surface will be inaccessible from the apical medium due to tight-junction formation. The present data show that the cellular vinblastine content, and the effect of inhibitors, depends upon whether the cells are grown upon plastic or a permeable matrix. In epithelial layers on a permeable matrix, vinblastine content also depends upon the surface from which the drug is presented; with basolateral presentation, cellular vinblastine is higher than with apical presentation (Table I). This implies that with a constant pump rate via P-glycoprotein, overall drug permeation via the basolateral membrane exceeds that of the apical surface due to differences in membrane area and/or lipid composition. Verapamil reduced cell-associated vinblastine when presented from the basal surface, with vinblastine also presented basally (Table I), presumably by reducing drug influx, since inhibition of P-glycoprotein mediated efflux alone would increase cell vinblastine. Verapamil was equally effective from either epithelial surfaces in inhibition of vectorial transepithelial vinblastine transport, suggesting that verapamil access to P-glycoprotein was not limited by apical or basolateral membranes.

The present data emphasise the potential importance of the expression of epithelial characteristics in intestinal adenocarcinoma cells towards an understanding of the biology of a tumour *in vivo*. P-glycoprotein function within an epithelial adenocarcinomal sheet generates net solute (vinblastine) flux consistent with its tissue of origin. Cellular accumulation of vinblastine within an epithelial sheet is not solely dependent upon P-glycoprotein function, and this study highlights the importance of differential membrane and junctional permeabilities (Figure 6). It is recognised that a variety of mechanisms are involved in mediating the modulation of MDR by chemosensitisers such as verapamil. In addition to inhibiting P-glycoprotein-mediated drug efflux, verapamil has also been described as inhibiting the ATP-dependent efflux of other xenobiotics such as bis-carboxyethyl-carboxyfluorescein (Allen *et al.*, 1990) and methotrexate (Sirotnak & O'Leary, 1991). In the present study our primary purpose has been to define P-glycoprotein-mediated flux in epithelial layers. The use of high concentrations of verapamil was necessitated by the requirement to inhibit maximally and rapidly P-glycoprotein pump activity. Non-specific effects of verapamil on epithelial function (e.g., intercellular junctional permeability) and cellular membrane permeability were noted (Figure 4b and 6), but these were not associated with acute toxicity assessed in the MTT assay. However, it is recognised that the chemosensitising action of verapamil is likely to be multifactorial, and may include perturbation of plasma membranes or lysosomal function, alterations in calcium homoeostasis, inhibition of other intracellular regulatory mechanisms (Ford & Hait, 1990), as well as interaction with P-glycoprotein.

We wish to thank the North of England Cancer Research Campaign for generous financial support for this work.

### References

ALLEN, C.N., HARPUR, E.S., GRAY, T.J.B. & HIRST, B.H. (1991). Toxic effects of non-steroidal anti-inflammatory drugs in a human intestinal epithelial cell line (HCT-8) assessed by the MTT and neutral red assay. *Toxicol. in Vitro*, **5** (in press).

ALLEN, C.N., HARPUR, E.S., GRAY, T.J.B., SIMMONS, N.L. & HIRST, B.H. (1990). Efflux of bis-carboxyethyl-carboxyfluorescein (BCECF) by a novel ATP-dependent transport mechanism in epithelial cells. *Biochem. Biophys. Res. Commun.*, **172**, 262.

BECK, W.T. (1987). The cell biology of multiple drug resistance. *Biochem. Pharmacol.*, **36**, 2879.

BELLAMY, W.T., DALTON, W.S., KAILEY, J.M., MCCLOSKEY, T.M., DORR, R.T. & ALBERTS, D.S. (1988). Verapamil reversal of doxorubicin resistance in multidrug-resistant human myeloma cells and association with drug accumulation and DNA damage. *Cancer Res.*, **48**, 6303.

BROXTERMAN, H.J., PINEDO, H.M, KUIPER, C.M. & 7 others (1989). Immunohistochemical detection of P-glycoprotein in human tumor cells with a low degree of drug resistance. *Int. J. Cancer*, **43**, 340.

CHAN, A.B., ALLEN, C.N., SIMMONS, N.L., PARSONS, M.E. & HIRST, B.H. (1989). Resistance to acid of canine kidney (MDCK) and human colonic (T84) and ileo-caecal (HCT-8) adenocarcinoma epithelial cell monolayers *in vitro*. *Quart. J. Exp. Physiol.*, **74**, 553.

CHANTRET, I., BARBAT, A., DUSSAULX, E., BRATTAIN, M.G. & ZWEIBAUM, A. (1988). Epithelial polarity, villin expression, and enterocytic differentiation of cultured human colon carcinoma cells: a survey of twenty cell lines. *Cancer Res.*, **48**, 1936.

CORDON-CARDO, C., O'BRIEN, J.P., BOCCIA, J., CASALS, D., BERTINO, J.R. & MELAMED, M.R. (1990). Expression of the multidrug resistance gene product (P-glycoprotein) in human normal and tumor tissues. *J. Histochem. Cytochem.*, **38**, 1277.

CORNWELL, M.M., SAFA, A.R., FELSTED, R.L., GOTTESMAN, M.M. & PASTAN, I. (1986). Membrane vesicles from multidrug-resistant human cancer cells contain a specific 150- to 170-kDa protein detected by photoaffinity labelling. *Proc. Natl Acad. Sci. USA*, **83**, 3847.

CORNWELL, M.M., PASTAN, I. & GOTTESMAN, M.M. (1987). Certain calcium channel blockers bind specifically to multidrug-resistant human KB carcinoma membrane vesicles and inhibit drug binding to P-glycoprotein. *J. Biol. Chem.*, **262**, 2166.

DANO, K. (1973). Active outward transport of daunmycin in resistant Ehrlich ascites tumor cells. *Biochem. Biophys. Acta.*, **323**, 466.

DEFFIE, A.M., ALAM, T., SENEVIRATNE, C. & 5 others (1988). Multifactorial resistance to adriamycin: relationship of DNA repair, glutathione transferase activity, drug efflux, and P-glycoprotein in cloned cell lines of adriamycin-sensitive and -resistant P388 leukemia. *Cancer Res.*, **48**, 3595.

DHARMSATHAPHORN, K., MCROBERTS, J.A., MANDEL, K.G., TISDALE, L.D. & MASUI, H. (1984). A human colonic tumour cell line that maintains vectorial electrolyte transport. *Am. J. Physiol.*, **246**, G204.

FOJO, A.T., UEDA, K., SLAMON, D.J., POPLACK, D.G., GOTTESMAN, M.M. & PASTAN, I. (1987). Expression of a multidrug-resistance gene in human tumors and tissues. *Proc. Natl Acad. Sci. USA*, **84**, 265.

FORD, J.M. & HAIT, W.N. (1990). Pharmacology of drugs that alter multidrug resistance in cancer. *Pharmacol. Rev.*, **42**, 155.

GOLDSTEIN, L.J., GALSKI, H., FOJO, A. & 11 others (1989). Expression of a multidrug resistance gene in human cancers. *J. Natl Cancer Inst.*, **81**, 116.

HAMADA, H., HAGIWARA, K.-I., NAKAJIMA, T. & TSURUO, T. (1987). Phosphorylation of the $M_r$ 170,000 to 180,000 glycoprotein specific to multidrug-resistant tumour cells: effects of verapamil, trifluoperazine, and phorbol esters. *Cancer Res.*, **47**, 2860.

HEYMAN, M., CRAIN-DENOYELLE, A.-M., NATH, S.K. & DESJEUX, J.-F. (1990). Quantification of protein transcytosis in the human colon carcinoma cell line CaCo-2. *J. Cell. Physiol.*, **143**, 391.

HORIO, M., CHIN, K.-V., CURRIER, S.J. & 5 others (1989). Transepithelial transport of drugs by the multidrug transporter in cultured Madin-Darby Canine Kidney cell epithelia. *J. Biol.. Chem.*, **264**, 14880.

HORIO, M., LOVELACE, E., PASTAN, I. & GOTTESMAN, M.M. (1991). Agents which reverse multidrug-resistance are inhibitors of [$^3$H]-vinblastine transport by isolated vesicles. *Biochem. Biophys. Acta.*, **1061**, 106.

HORTON, J.K., THIMMAIAH, K.N., HOUGHTON, J.A., HOROWITZ, M.E. & HOUGHTON, P.J. (1989). Modulation by verapamil of vincristine pharmacokinetics and toxicity in mice bearing human tumor xenografts. *Biochem. Pharmacol.*, **38**, 1727.

HUGHSON, E.J. & HOPKINS, C.R. (1990). Endocytoic pathways in polarised CaCo-2 cells: identification of an endosomal compartment accesible from both apical and basolateral surfaces. *J. Cell Biol.*, **110**, 337.

INABA, M., KOBAYASHI, H., SAKURAI, Y. & JOHNSON, K. (1979). Active efflux of daunorubicin and adriamycin in sensitive and resistant sublines of P388 leukemia. *Cancer Res.*, **39**, 2200.

KARTNER, N., EVERNDEN-PORELLE, D., BRADLEY, G. & LING, V. (1985). Detection of P-glycoprotein in multidrug-resistant cell lines by monoclonal antibodies. *Nature*, **316**, 820.

KEIZER, H.G., SCHUURHUIS, G.J., BROXTERMAN, H.J. & 5 others (1989). Correlation of multidrug resistance with decreased drug accumulation, altered subcellular drug distribution, and increased P-glycoprotein expression in cultured SW-1573 human lung tumor cells. *Cancer Res.*, **49**, 2988.

KESSEL, D. & WILBERDING, C. (1984). Mode of action of calcium antagonists which alter antracycline resistance. *Biochem. Pharmacol.*, **33**, 1157.

KESSEL, D. & WILBERDING, C. (1985). Promotion of daunorubicin uptake and toxicity by the calcium antagonist tiapamil and its analogs. *Cancer Treat. Report.*, **69**, 673.

KLOHS, W.D. & STEINKAMPF, R.W. (1988). Possible link between the intrinsic drug resistance of colon tumours and a detoxification mechanism of intestinal cells. *Cancer Res.*, **48**, 3025.

LAEMMLI, U.K. (1970). Cleavage of structural proteins during the assembly of the head of bacteriophage T4. *Nature*, **227**, 680.

LAMB, J.F., OGDEN, P. & SIMMONS, N.L. (1981). Autoradiographic localisation of $^3$Houabain bound to cultured epithelial cell monolayers of MDCK cells. *Biochem. Biophys. Acta*, **644**, 333.

MADARA, J.L. & DHARMSATHAPHORN, K. (1985). Occluing junction structure-function relationships in a cultured epithelial monolayer. *J. Cell Biol.*, **101**, 2124.

MADARA, J.L., STRAFFORD, J., DHARMSATHAPHORN, K. & CARLSON, S. (1987). Structural analysis of a human intestinal epithelial cell line. *Gastroenterology*, **92**, 1139.

NEDERMAN, T. & TWENTYMAN, P. (1984). Spheroids for studies of drug effects. *Recent Res. Cancer Res.*, **95**, 84.

PASTAN, I., GOTTESMAN, M.M., UEDA, K., LOVELACE, E., RUTHERFORD, A.V. & WILLINGHAM, M.C. (1988). A retrovirus carrying an MDR1 cDNA confers multidrug resistance and polarised expression of P-glycoprotein in MDCK cells. *Proc. Natl Acad. Sci. USA*, **85**, 4486.

PILLER, G.J. (1989). Leukaemia Research Fund International Research Symposium on cytotoxic drug resistance in leukemia and other malignancies. *Leukemia*, **3**, 461.

SAFA, A.R., GLOVER, C.J., MEYERS, M.B., BIEDLER, J.L. & FELSTED, R.L. (1986). Vinblastine photoaffinity labeling of a high molecular weight surface membrane glycoprotein specific for multidrug-resistant cells. *J. Biol. Chem.*, **261**, 6137.

SAFA, A.R., GLOVER, C.J., SEWELL, J.L., MEYERS, M.B., BIEDLER, J.L. & FELSTED, R.L. (1987). Identification of the multidrug-related membrane glycoproteins as an acceptor for calcium channel blockers. *J. Biol. Chem.*, **262**, 7884.

SCHEPER, R.J., BULTE, J.W.M., BRAKKE, J.G.P. & 8 others (1988). Monoclonal antibody JSB-1 detects a highly conserved epitope on the P-glycoprotein associated with multi-drug-resistance. *Int. J. Cancer*, **42**, 389.

SIMMONS, N.L. (1990). Tissue culture of established renal cell lines. *Meth. Enzymol.*, **191**, 426.

SIMONS, K. & FULLER, S.D. (1985). Cell surface polarity in epithelia. *Ann. Rev. Cell. Biol.*, **1**, 243.

SIROTNAK, F.M. & O'LEARY, D.F. (1991). The issues of transport multiplicity and energetics pertaining to methotrexate efflux in L1210 cells addressed by an analysis of *cis* and *trans* effects of inhibitors. *Cancer Res.*, **51**, 1412.

SKOVSGAARD, T. (1978*a*). Mechanisms of resistance to daunorubicin in Ehrlich ascites tumor cells. *Cancer Res.*, **38**, 1785.

SKOVSGAARD, T. (1978*b*). Mechanisms of cross-resistance between vincristine and daunorubicin in Ehrlich ascites tumor cells. *Cancer Res.*, **38**, 4722.

SUGAWARA, I., KATAOKA, I., MORISHITA, Y. & 4 others (1988). Tissue distribution of P-glycoprotein encoded by a multidrug-resistant gene as revealed by a monoclonal antibody, MRK 16. *Cancer Res.*, **48**, 1926.

THIEBAUT, F., TSURUO, T., HAMADA, H., GOTTESMAN, M.M., PASTAN, I. & WILLINGHAM, M.C. (1987). Cellular localization of the multidrug-resistance gene product P-glycoprotein in normal human tissues. *Proc. Natl Acad. Sci. USA*, **84**, 7735.

THIEBAUT, F., TSURUO, T., HAMADA, H., GOTTESMAN, M.M, PASTAN, I. & WILLINGHAM, M.C. (1989). Immunohistochemical localization in normal tissues of different epitopes in the multidrug transport protein P170: evidence for localization in brain capillaries and crossreactivity of one antibody with a muscle protein. *J. Histochem. Cytochem.*, **37**, 159.

THOMPSON, S. (1987). Simplified and reproducible silver staining of proteins in polyacrylamide gels. *Med. Sci. Res.*, **15**, 1253.

TOMPKINS, W.A.F., WATRACH, A.M., SCHMALE, J.D., SCHULTZ, R.M. & HARRIS, J.A. (1974). Cultural and antigenic properties of newly established cell strains derived from adenocarcinomas of the human colon and rectum. *J. Natl Cancer Inst.*, **52**, 1101.

TSURUO, T. (1983). Reversal of acquired resistance to vinca alkaloids and anthracycline antibiotics. *Cancer Treat. Report*, **67**, 889.

TWENTYMAN, P.R., FOX, N.E. & BLEEHEN, N.M. (1986). Drug resistance in human lung cancer cell lines: cross-resistance studies and effects of the calcium transport blocker, verapamil. *Int. J. Radiation Oncology Biol. Phys.*, **12**, 1355.

UEDA, K., CARDARELLI, C., GOTTESMAN, M.M. & PASTAN, I. (1987). Expression of a full length cDNA for the human 'MDR1' gene confers resistance to colchicine, doxorubicin, and vinblastine. *Proc. Natl Acad. Sci. USA*, **84**, 3004.

VAN DER BLIEK, A.M., BAAS, F., VAN DER VELDE-KOERTS, T. & 6 others (1988). Genes amplified and overexpressed in human multidrug-resistant cell lines. *Cancer Res.*, **48**, 5927.

VON BONSDORFF, C.-H., FULLER, S.D. & SIMONS, K. (1985). Apical and basolateral endocytosis in Madin-Derby canine kidney (MDCK) cells grown on nitrocellulose filters. *EMBO J.*, **4**, 2781.

WEINSTAIN, R.S., KUSZAK, J.R., KLUSKENS, J.R. & COON, J.S. (1990). P-glycoproteins in pathology: the multidrug resistance gene family in humans. *Human Pathol.*, **21**, 34.